# Longitudinal Syringomyelia, Cervical Dystonia, and Action Tremor in Trichorhinophalangeal Syndrome Type I – A Case Report

**DOI:** 10.5334/tohm.1116

**Published:** 2025-12-31

**Authors:** Bogdana Petko, Brent D. Weinberg, Jaime Vengoechea, Matthew Gary, Paul A. Beach

**Affiliations:** 1 Department of Neurology, Division of Movement Disorders, Emory University School of Medicine, Atlanta, Georgia, USA; 2 Department of Radiology and Imaging Sciences, Division of Neuroradiology, Emory University School of Medicine, Atlanta, Georgia, USA; 3 Department of Human Genetics, Division of Medical Genetics, Emory University School of Medicine, Atlanta, Georgia, USA; 4 Department of Surgery, Division of Neurosurgery, Emory University School of Medicine, Atlanta, Georgia, USA

**Keywords:** Syringomyelia, cervical dystonia, trichorhinophalangeal syndrome type I

## Abstract

**Background:**

Trichorhinophalangeal syndrome type I (TRPS I) is a rare, autosomal dominant disorder characterized by facial abnormalities, sparse hair, and skeletal deformities, including the skull base.

**Case Report:**

We report the case of a patient with TRPS I who was found to have syringomyelia and a movement disorder complex including action tremor and cervical dystonia.

**Discussion:**

Syringomyelia has been reported to occur in TRPS I secondary to posterior fossa abnormalities. We postulate that the patient’s cervical dystonia and action tremor are secondary to syringomyelia. The authors review possible mechanisms and review literature of similar cases.

**Highlights:**

This case report describes a patient with trichorhinophalangeal syndrome type I (TRPS I) with syringomyelia as well as action tremor and cervical dystonia. To our knowledge, this is the first report to demonstrate a triad of syringomyelia and two associated movement disorders with sequelae of TRPS I likely the ultimate cause.

## Introduction

Trichorhinophalangeal syndrome type I (TRPS I) is a rare, autosomal dominant genetic disorder caused by a heterozygous pathogenic variant in *TRPS1* on chromosome 8 with about one-third occurring *de novo* [1,2]. This gene encodes the Zinc finger transcription factor Trps1 (TRPS1 protein), which plays a role in the development of cartilage, bone, and hair follicles [3]. The disorder is characterized by facial abnormalities, sparse and slow-growing scalp hair, premature baldness, laterally sparse eyebrows, bulbous pear-shaped nose, elongated and flat philtrum, thin upper lip, and protruding ears [4]. Skeletal abnormalities include cone-shaped epiphyses, clinodactyly, short stature, and hip joint malformations [5]. Bony defects may also include skull base abnormalities, which can lead to posterior fossa abnormalities and cerebellar tonsillar displacement, which in turn may contribute to development of syringomyelia [6,7].

Syringomyelia, which is frequently associated with Chiari I deformity, is characterized by the formation of a fluid-filled cyst, or syrinx, within the cervical or thoracic spinal cord [8]. Syringomyelia typically impacts neurologic function in the arms and hands, leading to bilateral weakness and deficits in temperature and pain sensation, with intact vibration and proprioception [9]. Syringomyelia has also been implicated in the etiology of movement disorders, including dystonia, myoclonus, tremor, and athetosis [10,11]. Treatment includes conservative, symptomatic management and, in refractory and disabling cases, surgical intervention. For patients with Chiari I deformity, cranio-cervical decompression is the first line surgical treatment, followed by CSF shunting [8].

Cervical dystonia (CD) is a neurological disorder characterized by involuntary, repetitive head and neck movements [12]. Several neuroanatomical regions are implicated in CD pathophysiology, including the cerebellum, midbrain, and prefrontal, motor, and somatosensory cortices [12]. The involvement of these diverse anatomical regions has led to the recognition of dystonia as a sensorimotor network disorder [13]. Jerky hand tremor in the setting of CD and progressively worsening head tremor have been described in patients with syringomyelia [11,14].

Here we present a patient with a genetic diagnosis of TRPS I whose mutation was discovered after workup for CD in the setting of chronic action tremor, facial dysmorphia, joint hyperextensibility, and a longitudinal syringomyelia of unclear etiology. To our knowledge, this is the first case report on a triad of syringomyelia, action tremor and CD occurring in a patient with TRPS I. We discuss the possible pathogenic mechanisms involved in this patient’s condition and provide a brief review of similar cases reported in literature.

## Case Report

*History and Presentation*. A 31-year-old female with a medical history notable for attention-deficit/hyperactivity disorder, anxiety, depression, chronic low back and shoulder pain was referred for evaluation of tremor. She reported development of bilateral upper extremity action tremor between the ages of 11 and 13, later diagnosed as essential tremor (ET). The tremor progressively worsened, affecting her feet and voice as well as her hands. In addition, she reported occipital pain that radiated to thoracic, bilateral scapular, and upper arm regions with associated ‘patches’ of numbness in the arms and scapular regions that had occurred since childhood. Her family history included action tremor on the paternal side, affecting her father, paternal uncle, and paternal grandmother. She did not have a known family history of genetic mutations, including TRPS I.

*Examination*. Neurological examination revealed CD characterized by mild right laterocaput, mild left torticollis, left shoulder elevation, and hypertrophy of the right sternocleidomastoid (Video 1). She did not exhibit parkinsonism, and had a low amplitude, jerky postural and action tremor in both hands, predominantly with intention. She reported mild hand cramping while writing. She had normal strength with relative hypotonia and normal sensation to pinprick, cold temperature, and vibration. General examination revealed mild asymmetric facial dysmorphia with low-set ears, smooth philtrum, thin upper lip, and retrognathia. She had a Beighton score of 8/9 (highly suggestive of hypermobility) and slightly dysmorphic hands.

**Video 1:** **Video examination of patient with TRPS I cervical dystonia and action tremor.** Patient with mild right laterocaput, mild left torticollis, and left shoulder elevation. She had slight restriction of movement with left head turn. She had mild, bilateral low-amplitude slightly jerky action tremor worse with intention as well as mild postural tremor. Writing revealed mild tremor on spirals, but handwriting was otherwise without tremor. Gait was generally normal, but showed left shoulder elevation. Retrognathia, low set ears, and joint hyperextensibility also noted.

**Figure 1:**
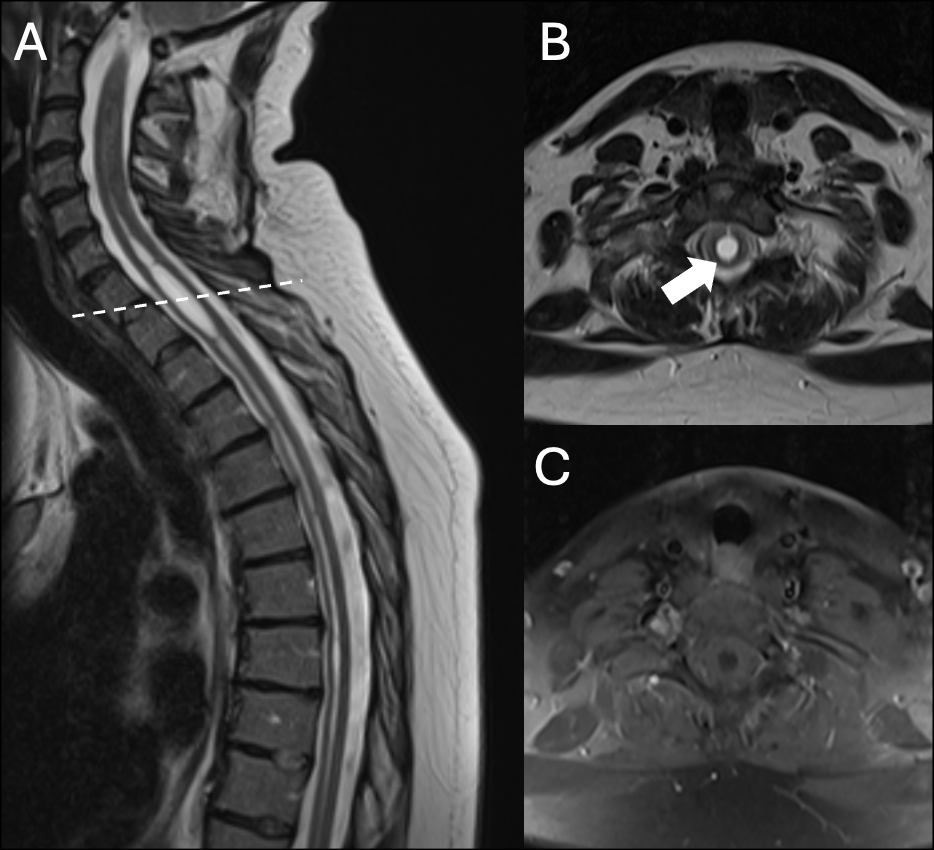
**A.** Sagittal T2 image of the thoracic spine showing syringomyelia extending from the mid cervical spine through the lower lumbar spine. Dashed line shows the level of accompanying axial images. **B.** Axial T2 image shows a syrinx in the middle of the spinal cord (white arrow). **C.** Axial postcontrast T1 image showed no abnormal enhancement.

**Figure 2:**
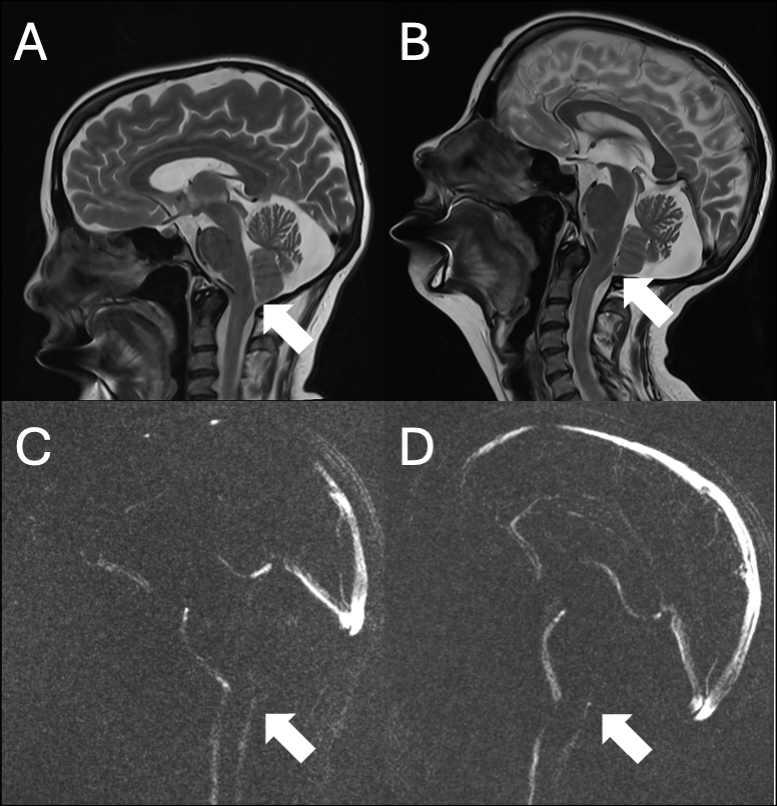
**A.** Sagittal T2 image of the craniocervical junction with the head in flexion showing low-lying cerebellar tonsils with crowding of the foramen magnum (arrow). **B.** Sagittal T2 image showing worsening crowding with extension. **C.** Phase contrast flow image in flexion showing diminished magnitude of flow in the dorsal foramen magnum. **D.** Phase contrast flow in extension showing worsening of decreased flow in the dorsal foramen magnum.

*Diagnostics*. She was diagnosed with CD, possible segmental involvement of the arms, resembling a mild writer’s cramp, and superimposed action tremor. Brain and spinal MRI were ordered as part of the diagnostic work-up due to this movement disorder complex in a young patient (Figure 1). A cervical spine MRI without contrast identified a longitudinal syringomyelia from C5 to T11. MRI Brain revealed low-lying cerebellar tonsils not meeting criteria for Chiari deformity and mild prominence of the midline posterior extra-axial space, likely representing a mega cisterna magna versus a small arachnoid cyst. Follow-up scanning of the brain and craniocervical junction, including thin slice sagittal T2 images and phase contrast cine flow images, was performed with the head in flexion and extension (Figure 2). These images showed decreased bidirectional flow in the dorsal foramen magnum that worsened with the upper cervical spine in extension. These findings suggested altered CSF dynamics at the foramen magnum that may have contributed to syrinx formation. She was referred for neurosurgery and genetics consultations.

Trio whole exome sequencing identified a maternally inherited heterozygous pathogenic frameshift variant in *TRPS1*, c.2343_2344del. Coupled with her physical features, a diagnosis of TRPS I was established. Notably, the lab did not report variants in dystonia-related genes on the trio exome. During neurosurgical evaluation, surgical intervention was initially not advised as her symptoms were largely sensory; she had subjective weakness in lower extremities, but no focal weakness on initial examination. However, over the following year she developed worsening motor strength (4+/5 on the Medical Research Council, MRC) and required a walker for gait assistance. Ultimately, the decision was made to proceed with surgery given clinically evident myelopathy.

*Surgical Intervention and post-operative course*. A suboccipital craniectomy with C1 laminectomy for Chiari decompression was performed due to the patient’s progressive cervico-thoracic syringomyelia symptoms and suspected etiology at the level of the foramen magnum. Post-operatively, she experienced mild improvement in motor and sensory symptoms and was initially able to ambulate without a walker, though she continued to experience chronic neck and back pain. Three years post-surgery, she continued to report left-sided numbness and tingling and gait difficulties necessitating the use of a cane.

Post-surgical MRI showed cranio-cervical junction decompression with a wide CSF space dorsal to the lower brain stem and upper cervical cord. The cervical-thoracic syrinx was still present but had decreased in size.

*Medical treatment*. Prior to our evaluation of the patient, she had been treated with several medications for a presumed diagnosis of ET. Primidone 50 mg twice daily (BID) was excessively sedating. Topiramate 50 mg BID, zonisamide (unknown dose), clonazepam 0.5 mg BID, and methocarbamol 1000 mg three times daily (TID) were ineffective. Propranolol 60 mg BID provided only a transient benefit. IncobotulinumtoxinA 200 units in unknown dosing pattern for CD offered transient, mild relief of neck and shoulder pain.

## Discussion

Here we report a case of TRPS I associated with syringomyelia and suspected secondary CD and action tremor. While case reports exist of TRPS I and syringomyelia, as well as associations between syringomyelia and movement disorders, such as tremor or dystonia, this is the first case report to demonstrate a triad of syringomyelia and two associated movement disorders with TRPS I likely as the ultimate cause.

Previous case reports have documented patients with TRPS I and syringomyelia. Fernandez (1993) reported a case of a boy with TRPS I and syringomyelia in the context of basilar impression, characterized by the displacement of the foramen magnum into the posterior cranial fossa and upward displacement of the cervical spine [6]. Another case report by Martinez-Lage (2008) described a patient with TRPS I, Chiari I deformity with associated syringomyelia, and an arachnoid cyst [15]. The gene *TRPS1* is involved with endochondral ossification, the main developmental mechanisms at the skull base. Thus, in these previously reported cases and in our own, these developments can be attributed to abnormal skull base softening, abnormal posterior fossa development, tonsillar displacement, and arachnoid cyst formation [15]. No prior case report described dystonia (or any other movement disorder) on physical examination.

In addition to causing typical bilateral motor and sensory symptoms, syringomyelia is associated with several movement disorders [11]. Action and postural tremor resembling ET or physiologic tremor, spinal myoclonus, and dystonia-athetosis have been reported in patients with syringomyelia [10]. Clinical reports of improvement of dystonia after Chiari decompression treatment also support the notion of a direct link between syringomyelia and CD [11]. Although most cases of syringomyelia do not result in dystonia, dystonia in the setting of syringomyelia may be underdiagnosed, as spinal imaging is not commonly performed in dystonia evaluations.

The pathophysiology of syringomyelia-associated dystonia remains uncertain. Mulroy et al (2019) proposed three possible mechanisms for syringomyelia-associated dystonia: abnormal sensorimotor integration, loss of spinal motor pathway inhibition, and cerebellar dysfunction [11]. The motor system generates a prediction of sensory consequences of a planned movement [16]. Dysfunctional sensory tracts from syringomyelia-based structural disruption, may lead to inaccurate sensory feedback and dysfunctional corrective motor outputs that manifest clinically as dystonia [16,17]. Syringomyelia may additionally interrupt inhibitory interneuron activity at the spinal cord, leading to co-contraction of agonist and antagonist muscles and subsequent dystonia [11]. At the cerebellar level, Chiari I deformity is associated with alterations in cerebellar connectivity [18]. For our patient, it is thus also possible that a combination of structural spinal cord dysfunction as well as low-lying cerebellar tonsils with altered CSF dynamics at the foramen magnum contributed to dystonia through a such a mechanism.

Our patient’s posterior fossa abnormalities likely resulted from skull base weakening through abnormal endochondral ossification as seen in TRPS I. While this patient did not have a Chiari I deformity, there was decreased CSF flow in the dorsal foramen magnum, a sign of obstructed CSF flow. This finding had a positional component, being worse in extension, that may have further contributed to subsequent syringomyelia. We propose that her CD developed secondary to syringomyelia, potentially due to processes such as impaired spinal inhibitory and sensorimotor integration mechanisms as described above. She reported sensory symptoms since childhood, so it is possible she had syringomyelia for years prior to its discovery, as no spinal imaging occurred prior to our evaluation. It is unclear when her cervical dystonia developed, as this was first noted at our initial evaluation. Additionally, although she has a family history of tremor, diagnosed as ET, given the slightly atypical features of her hand tremor with jerky characteristics, it is possible that the tremor was one of the first manifestations of syringomyelia as well.

The optimal management in dystonia secondary to syringomyelia remains uncertain. For our patient, her CD was less debilitating in comparison to the weakness and sensory deficits. Following surgery, she continued to decline in mobility and now uses a cane. The dystonia has also persisted without significant response to botulinum toxin.

## Conclusion

We have reported a case of TRPS I with syringomyelia and suspected secondary action tremor and CD. Reports of an association between TRPS I and dystonia have never previously been reported, and further case reports or case series are needed to further understand underlying pathophysiology. Nervous system expression of the gene TRPS1 is limited to astrocytes, and deletions in the gene in rodent models affected cortical astrocytic synapse maintenance and neuroimmune interactions [19]. However, it is unclear if the mutation causing TRPS I in humans causes any primary neurologic manifestations. Based on this, we propose that our patient’s movement disorder complex is secondary to syringomyelia, which occurred due to the known effects TRPS I may have on the skull base. While we cannot rule out cerebellar dysfunction, this mechanism seems less likely as the only prior case report mentioning a structural abnormality of the nervous system – a Chiari 1 malformation – did not discuss neurologic symptoms in that patient. Genetic and phenotypic characterization (including neuroimaging) of patient family members with tremor would also help clarify mechanism of our patient’s presentation. However, information beyond simple family history was not available to us. Overall, our case report notes the importance of ordering cervical spine imaging in atypical cases of CD and highlights links between spinal pathology and movement disorders.
